# Numerical and Experimental Study on the Development of Electric Sensor as for Measurement of Red Blood Cell Deformability in Microchannels

**DOI:** 10.3390/s120810566

**Published:** 2012-08-03

**Authors:** Kazuya Tatsumi, Yoichi Katsumoto, Ryoji Fujiwara, Kazuyoshi Nakabe

**Affiliations:** Department of Mechanical Engineering and Science, Kyoto University, Sakyo-ku, Kyoto 606-8501, Japan; E-Mails: yoichi.katsumoto@fy7.ecs.kyoto-u.ac.jp (Y.K.); ryouji.fujiwara@t06.mbox.media.kyoto-u.ac.jp (R.F.); nakabe@me.kyoto-u.ac.jp (K.N.)

**Keywords:** red blood cell, deformability, microchannel, electric sensor, laminar flow, shear stress, Ca^2+^ ionophore treatment, Micro-TAS

## Abstract

A microsensor that can continuously measure the deformability of a single red blood cell (RBC) in its microchannels using microelectrodes is described in this paper. The time series of the electric resistance is measured using an AC current *vs.* voltage method as the RBC passes between counter-electrode-type micro-membrane sensors attached to the bottom wall of the microchannel. The RBC is deformed by the shear flow created in the microchannel; the degree of deformation depends on the elastic modulus of the RBC. The resistance distribution, which is unique to the shape of the RBC, is analyzed to obtain the deformability of each cell. First, a numerical simulation of the electric field around the electrodes and RBC is carried out to evaluate the influences of the RBC height position, channel height, distance between the electrodes, electrode width, and RBC shape on the sensor sensitivity. Then, a microsensor was designed and fabricated on the basis of the numerical results. Resistance measurement was carried out using samples of normal RBCs and rigidified (Ca^2+^–A23186 treated) RBCs. Visualization measurement of the cells' behavior was carried out using a high-speed camera, and the results were compared with those obtained above to evaluate the performance of the sensor.

## 1. Introduction

A microsensor that can efficiently and sequentially measure the deformability of individual red blood cells (RBCs) on the basis of microchannel flows is described in this paper. The use of a microchannel offers advantages such as reduced cost, measurement time, and sample volume. Furthermore, measuring the deformability of each RBC suspended in a solution can markedly increase the accuracy of the measurement. Our results will make a significant contribution to various fields of medicine in terms of detection of diseases in the early stages [1–5]. Further, the present method measuring the cell deformability can be applied to detect the activity of the leukocyte [6].

Currently, several types of methods are typically used to measure the deformability of RBCs. Among them, relatively simple ways to measure the deformability of cells involve the use of viscometers and rheometers. In the case of a viscometer, the cell deformability is measured by considering the fluid viscosity [7]. A rheometer is used to measure the deformability of an RBC by applying shear stress to it in a coaxial rotating cylinder and then visually analyzing the shape of the cell [8]. Because many cells are suspended in the solution that is used in these measurements, cell deformability or its effects are measured as the average of all the cells. Thus, these methods are useful for determining the relationship between cell deformability and fluid properties or characteristics. However, when the number of cells exhibiting different deformability is small, the accuracy of these methods reduces to levels that are undesirable in clinical testing for diseases that are still in the early stages. For example, Plasmodium falciparum, a highly infectious parasite that causes severe anemia in a number of tissues and organs [1], considerably reduces the deformability of RBCs by producing cytoadherence-related neoantigens; these antigens increase the internal viscosity and rigidity of the cytomembrane [2,3]. The deformability of these RBCs can be analyzed to diagnose such diseases. However, as the number of influenced cells in the blood is very small, sensors with very high sensitivity are required.

There are several measurement methods that can evaluate the deformability of a single RBC [9]. In the micropipette aspiration technique, the deformability of the aspirated cells is measured by considering their deformation rate and aspiration pressure [10,11]. Another common method is to use microtweezers to stretch a cell by applying force to beads that are attached to both ends of the cell [3,12,13]. Other new methods involve the use of electric force to tense the RBC [14] or measurement of the unsteady behavior of the RBC in shear flows [15]. The accuracy of these measurement methods is much higher than that of the previously mentioned bulk fluid measurement methods. However, as the number of cells that are available for measurement is quite small, these methods are not suitable for clinical diagnoses.

Microchannels and microsensors have recently come to be considered as powerful tools in clinical diagnoses. The use of microsensors offers not only the advantages mentioned earlier, but also an effective solution to problems encountered in the measurement of RBC deformability with a high accuracy. Tracey *et al.* [16] fabricated a microchip with several embedded microchannels having a width of 4 μm and visually measured the deformation rate of cells that passed through the microchannels. Korin *et al.* [17] proposed an interesting method to visualize the behavior of RBCs in a microchannel; the cell deformation is measured by analyzing their images as they are stretched by high shear flows. However, this method requires high-resolution image-recording equipment that is bulky and expensive.

**Figure 1.**
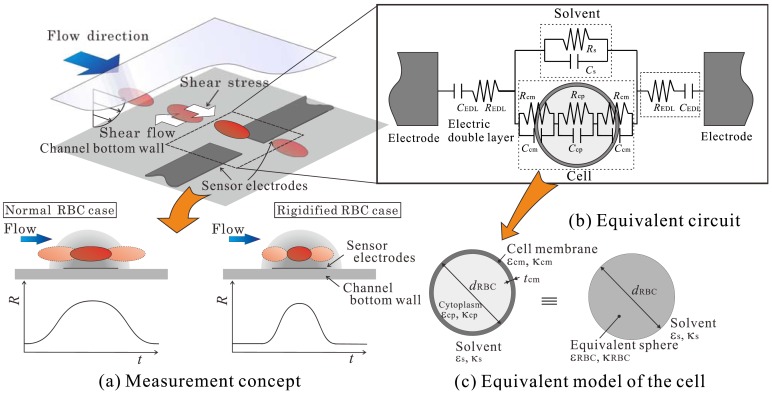
(**a**) Schematic views of the proposed method for measuring the RBC deformability; (**b**) equivalent circuit of the region between the electrodes; (**c**) equivalent model of a cell considered as a sphere with uniform complex permittivity.

The microsensor proposed in this study measures the electric resistance of RBCs as they pass between its electrodes. Figure 1 shows a schematic of the basic elements of the proposed sensor. As shown in Figure 1(a), micro-membrane-type electrodes are attached to the bottom wall of the microchannel. An RBC is suspended in the flow and is controlled such that it passes between the electrodes. An equivalent circuit that represents the electric characteristics of the region between the electrodes is shown in Figure 1(b). The circuit consists of the cell, a solution, and an electric double layer formed on the surface of the electrodes. The cell is composed of the cytoplasm and cytomembrane. The resistance of the cytomembrane is much larger than that of the solution and is less than 1 × 10^–6^ S/m. This value is less than that of the cytoplasm and normal saline solution; thus, the cytomembrane acts as an insulating material. In this case, the resistance obtained from the impedance measured by the electrodes will be influenced mainly by two factors: that are, the resistance of the membrane and how the current flux in the electric field is interruption by the cell. This means that the measured resistance will reflect the size, height position, and shape of the cell. In addition to this, when the RBC passes the electrodes, the resistance will increase as it approaches the center of the electrodes and then decreases as it moves away; namely, the resistance will show a time-series distribution similar to the one shown by the graph in Figure 1(a). The previously mentioned parameters are considered to not only influence the resistance itself but also this time-series distribution of the resistance.

As shown by the schematic in Figure 1(a), the RBC is suspended in a microchannel shear flow by which a constant shear stress will be applied to the cell. In this case, the deformation degree of the RBC, in other words the shape of the RBC, will rely on the deformability of the cell. Considering then that the RBC shape is one of the factors that influence the resistance and its distribution measured by the electrodes, this means that if the height position of RBC can be precisely controlled, the RBC deformability can be measured by analyzing the resistance and its time-series distribution.

On the basis of these physical concepts, we have fabricated a microsensor consisting of a microchannel and electrodes [18]. Measurements were conducted upon two kinds of samples, normal human RBCs and glutaraldehyde-treated (rigidified) RBCs, to evaluate the feasibility of the sensor. Further, the influence of the applied voltage frequency on the resistance of the RBC and electric double layer formed at the electrode surfaces were evaluated in order to specify the certain frequency that can effectively increase the sensor sensitivity. In this paper, we will first carry out numerical simulations of the electric field around the symmetric sensor electrodes when the RBC passes between them. The effects of the cell height position, channel height, and size of the electrodes are evaluated in order to provide some insight into the optimum shape of the sensor to be used in the experiment. The symmetric electrode-type sensor is then fabricated on the basis of the numerical results. The performance of the sensor is evaluated experimentally by using samples of normal human RBCs and rigidified RBCs. In preparing the rigidified RBC, the Ca^2+^ concentration in the RBC is controlled by using an ionophore rather than using glutaraldehyde. This way enables us to rigidify the RBC under the condition similar to the one of the actual phenomena *in vivo*, and also to gradually control the deformability of the RBC. The behavior of the RBCs will be visualized simultaneously using a high-speed video camera. The results are then compared with those obtained by the electrical measurements to determine the relationship between the resistance distribution and the deformation rate of individual RBC, and the performance of the proposed sensor is evaluated.

## 2. Numerical Methods

Numerical simulation was carried out to evaluate the impedance characteristics between the electrodes when the cell passes between them. Several parameters representing the sensor geometry were varied and their effects on the sensor's sensitivity were examined to gain insights that would ensure the design of an optimally shaped sensor. The computation was carried out employing commercial software (ANSYS ver. 11: ANSYS Inc.). Using the finite element method, the three-dimensional steady harmonic electric field in the area that includes the microchannel, channel walls, sensor electrodes, and RBC was calculated by solving the following governing equation for the electric potential *V*:

(1) $$\nabla \cdot \left(\left(\varepsilon -i\frac{\kappa }{\varepsilon_{0}\omega }\right)\nabla V\right)=0$$

where *ε* is the relative permittivity, *ε*_0_ is the vacuum permittivity, and *κ* is the conductivity. *ω* is the angular frequency.

### 2.1. Cell Model

As described in Section 1, the electrical properties of the RBC are mainly attributed to the cytoplasm and cytomembrane (see Figure 1(b)). However, the membrane thickness is of the order of nanometers and is extremely small as compared with the size of the channel and cytoplasm. Thus, if the size of the mesh employed in the computation is defined on the basis of the membrane thickness, the total number of grids and the resultant computational load increase considerably. To tackle this problem, the cell is modeled as a sphere with a uniform complex permittivity ${\text{ε}}_{\text{RBC}}^{*}$, as shown in Figure 1(c). Using this approach, it is possible to markedly decrease the number of grids without diminishing the accuracy of measurement of the RBC's electrical characteristics [19]. The variable ${\text{ε}}_{\text{RBC}}^{*}$ was defined by Hanai [20], as shown in Equation (2):

(2) $$\varepsilon_{\text{RBC}}^{*}=\varepsilon_{cm}^{*}\frac{2(1-\nu )\varepsilon_{cm}^{*}+(1+2\nu )\varepsilon_{cp}^{*}}{(2+\nu )\varepsilon_{cm}^{*}+(1-\nu )\varepsilon_{cp}^{*}}$$

where $\nu =\frac{d_{\text{RBC}}-2t_{cm}}{d_{\text{RBC}}}$, *d*_RBC_ is the diameter of the sphere, and *t*_cm_ is the membrane thickness. The complex permittivity of the cytoplasm and the membrane, ${\text{ε}}_{\text{cp}}^{*}$ and ${\text{ε}}_{\text{cm}}^{*}$, respectively, are calculated by applying the relative permittivity and conductivity of the cytoplasm, ***ε_cp_, k_cp_***, and the relative permittivity and conductivity of the membrane ***ε_cm_***, and ***k_cm_***, respectively, to Equation (3):

(3) $$\varepsilon^{*}=\left(\varepsilon -i\frac{\kappa }{\varepsilon_{0}\omega }\right)$$

### 2.2. Computational Conditions

**Figure 2.**
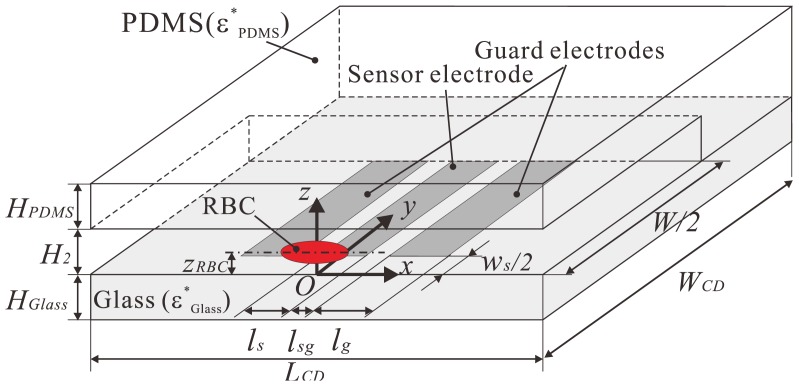
Computational domain of the numerical simulations.

The computational domain is shown in Figure 2. Since the sensor electrodes have a symmetric pattern with respect to the centerline of the channel, a symmetric configuration was applied to the *x-z* plane in the middle of the electrodes. The material of the bottom wall was glass with a thickness of *H*_Glass_. The upper and side walls were made of PDMS, with properties of *ε*_Glass_ = 3.4, *κ*_Glass_ = 1.0 × 10^−12^ S/m, *ε*_PDMS_ = 2.5, and *κ*_PDMS_ = 1.0 × 10^−12^ S/m, respectively. The distance between the RBC center and the bottom wall *z*_RBC_, the channel height *H*_2_, the distance between the sensor and ground electrodes *w*_s_, and the sensor width *l*_s_, were varied in the computation and will be discussed independently. Other variables were kept constant during the computation and are summarized in Table 1.

The thickness of the electrode is zero. The electric potential of *V*_add_ [V] was applied to the surface of the electrodes. On the other hand, an electric potential of 0 V was applied to the remaining boundaries of the computational domain, including the *x-z* plane at *y* = 0 (the symmetric boundary). The frequency of the applied AC voltage *V*_add_ was 10 kHz, which is the same as that applied to the electrodes in the experiment. The complex current measured at each grid node in the region between the electrodes was divided by 2*V*_add_ to obtain the complex impedance. The electric resistance between the electrodes was then derived from the parallel circuit shown in Figure 1(b).

The RBC was considered to be an ellipsoidal body (*a* = long axis, *b* = short axis) with the long axis aligned in the streamwise direction. The ratio of *a* and *b* was changed in order to express different deformations of the RBC. In this case, while *a* and *b* were varied, the surface area was kept constant [21]. The diameter of the RBC was *d*_RBC_ = *a* = *b* = 6.7 μm when the RBC was regarded as a sphere. The degree of deformation will be discussed by using the deformation index *DI* [17], as shown in Equation (4):

(4) $$DI=\frac{a-b}{a+b}$$

## 3. Materials and Methods

### 3.1. Microchannel and Electrodes

**Figure 3.**
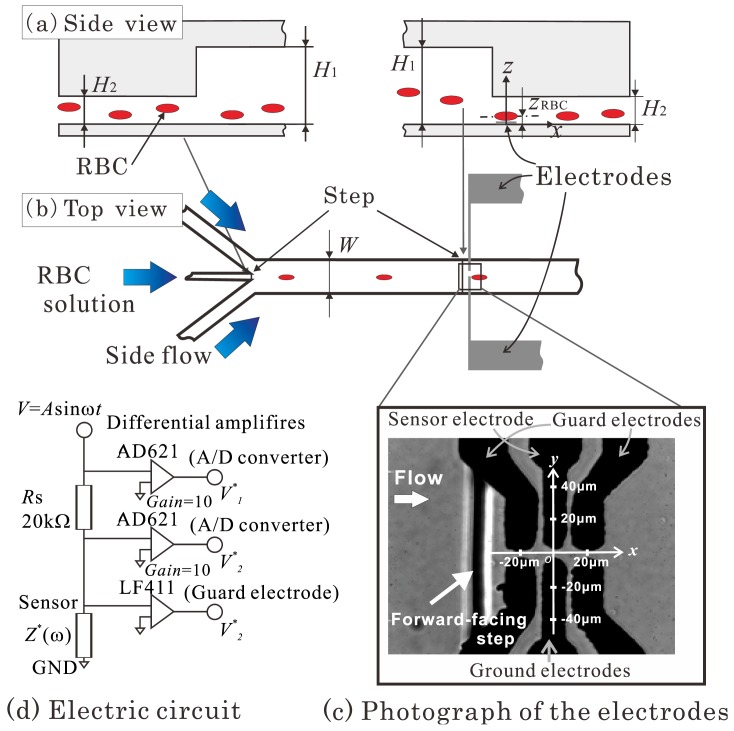
(**a**) and (**b**) schematics of the microchannel and electrodes of the sensor used in the experiment; (**c**) photograph of the electrodes used in the experiment; (**d**) schematic of the sensor electric circuit.

Figure 3(a,b) shows a schematic diagram of the channel used in the experiment. The channel width, *W*, is 1 mm, which is much larger than the channel heights *H*_1_ and *H*_2_. The RBC, therefore, can be considered to deform on experiencing the shear stress from a two-dimensional channel flow. Three inlets were located upstream of the channel. RBCs suspended in solution were supplied from the central inlet, while the solution alone was supplied from the other two side flows. A sheath flow was thus generated in the channel that could guide the RBCs to the region between the electrodes.

When a RBC is placed in a channel shear flow, the cell receives a lift force that is attributed to the so-called Fåhraeus effect [22]. This lift force moves the RBC away from the channel wall on which the sensor electrodes are embedded, which apparently leads to a decrease in both the measuring resistance and the accuracy of the sensor. To tackle this problem, a backward-facing step was placed at the point where the central inlet and the main channel come together, and a forward-facing step was placed immediately upstream of the electrodes, as shown in Figure 3(a). The RBC supplied from the central inlet would thus remain in a lower position in the main channel, and the downwash flow at the forward-facing step would carry the RBC downward, closer to the bottom wall of the channel. The combined effects of these structures enabled us to control the RBC height position, *z*_RBC_, so that the RBC would be close to the electrodes (*z*_RBC_ ≅ 2 μm) when it passed between them.

The microchannel was made of polydimethylsiloxane (PDMS, Shin-Etsu Chemical Co. Ltd.; KE-106) and was fabricated using SU-8 (MicroChem Co.) as a casting mold. The PDMS channel, which was removed from the SU-8, was attached to a glass slide, on which the platinum membrane electrodes were patterned.

Figure 3(c) shows a photograph of the electrodes. The flow direction is from left to right, and the vertical black line represents the forward-facing step. Counter-type platinum membrane electrodes were transversely and symmetrically attached to the bottom wall of the channel. Guard electrodes were placed on both sides of the sensor electrode. An electrical potential identical to that applied to the sensor electrode was supplied to these guard electrodes in order to reduce the fringe effect of the electric field observed at the edges of the sensor electrode. This led to an increase in the sensitivity of the sensor.

The platinum electrodes were fabricated through sputtering and lift-off processes using an Electron Beam Lithography system, a sputter device (ULVAC KIKO Inc.; SCOTT-C3), and photoresist apparatus (ZEON Co.; ZPN). It should be noted that in order to obtain a stronger contact between the platinum and glass, a contact layer of chrome was sputtered onto the glass slide before the platinum layer was formed. The thickness of these layers was on the order of hundreds of nm, as measured by a surface profiler.

In order to increase the microscopic surface area of the electrodes to reduce the impedance of the electric double layer formed on them when the voltage is supplied, the electrodes were plated with platinum nanoparticles. This accounts for the relatively rough edges of the electrodes shown in Figure 3(c). The plating process took place as follows. First, the electrode was washed by filling the channel with 0.5 M sulfuric acid for 10 min. The channel was then washed by running pure water through it. The channel was then filled with a solution of 30 mg/mL hexachloroplatinic acid (H_2_PtCl_6_) and 0.3 mg/mL lead acetate [(CH_3_COO)_2_Pb]. One V DC voltage was applied to the electrode, and the platinum nanoparticles were plated onto its surface. The channel and electrode were then washed with pure water. After being filled with 0.5 M sulfuric acid for 10 min, the channel was again washed with pure water.

### 3.2. Electrical Resistance Measurement

The circuit used in this study to measure the resistance between the electrodes is shown in Figure 3(d). The shunt resistor of *R*_s_ = 20 kΩ for current detection was connected serially to the sensor electrode whose opposite side was grounded. A sinusoidal wave of AC voltage with an amplitude of 1 V_p-p_ and a frequency of 10 kHz was applied to the upper part of the shunt resistor using a function generator (NF Co.; WF-1973). The guard electrodes were connected to the sensor electrode through a JFET input op-amp (National Semiconductor Co.; LF411), and the potential of these electrodes was kept identical to that applied to the sensor electrode. The upper port potential *V*_1_ and the sensor electrode potential *V*_2_ were impedance-converted by an op-amp (Analog Devices Inc.; AD621).

The signals *V*_1_ and *V*_2_ were simultaneously recorded by a computer through an A/D board (National Instruments Co.; PCIe-6251) with a sampling frequency of 600 kHz. After measurement, *V*_1_ and *V*_2_ were analyzed to obtain the magnitude and phase of each signal. *V*_1_*, which represents the complex values of *V*_1_ and is the original voltage signal, was subtracted from *V*_2_*, which represents the complex value of *V*_2_. The complex impedance between the sensor and the ground electrodes, Z*, could then be obtained by dividing *V*_2_* by *V*_1_*−*V*_2_* and multiplying it by the shunt resistance, as shown in Equation (5):

(5) $$Z^{*}(\omega )=R_{s}\frac{V_{2}^{*}}{V_{1}^{*}-V_{2}^{*}}$$

By this procedure, the influence of the electrical noises superposed on the signals could be effectively reduced. The resistance between the electrodes, *R*, was then obtained from Z*.

### 3.3. Visualization Measurement

Simultaneous with the above electrical measurements, visualization measurement were carried out in order to verify the shape and position of the RBCs when they passed the forward-facing step and the electrodes. This measurement was carried out using an inverted-type microscope (Olympus Co.; IX-71) with a halogen lamp as the light source. The objective lens was a long working distance lens with ×100 magnification and a numerical aperture of *NA* = 0.8 (Olympus Co.; LMPLFLN100X). The images were recorded by a high speed camera (Vision Research; Phantom V7.3). The frame rate of the camera was 1,000 frames/s and the CCD resolution was 800 × 650 pixels. The optical resolution of the image in combination with the lens was 0.073 μm/pixel. The focusing depth of the image was 1.7 μm [23].

### 3.4. Preparation of Red Blood Cells

A small amount of sample blood was collected from healthy human volunteers (the operator). To prepare the normal RBCs, the blood was first washed twice via the following procedure: the blood was suspended in phosphate buffered saline (PBS, Amresco Inc.; E404) solution to which 0.019 mM adenosine 5′-triphosphate disodium salt (ATP, Oriental Yeast Co. Ltd.) was added, and the pH was controlled by HCl to be 6.9. The solution was centrifuged for 10 min at 2,000 G, and the supernatant was removed. The precipitated RBCs were collected and suspended in PBS with 10 wt% polyvinylpyrrolidone (PVP, MW = 3.6 × 10^5^, Nacalai Tesque; K-90).

Rigidified RBCs were prepared by treating normal RBCs with ionophore (A23182, Alomone Labs) and increasing the calcium concentration of the cytoplasm [24–26]. These RBCs were first washed twice in the same way as the normal RBCs. The precipitated RBCs were then suspended in a Tris solution (1 mol/L Tris-HCl buffer solution, pH = 7.6; Nacalai Tesque) with Ca^2+^ and A23187 (KCl: 10 mM, NaCl: 130 mM, MgCl_2_: 2 mM, Tris: 15 mM, CaCl_2_: 1 mM, A23187: 1 mM, ATP: 0.48 mM). Commercial A23187 in powder form was used. A23187 was, therefore, first dissolved in an ethanol and water (1:3) solution, then mixed with the previously mentioned solution. The solution was heated at 37 °C for 40 min. After cooling to room temperature, the solution was centrifuged and the supernatant was removed. The RBCs were then washed twice with PBS. The collected RBCs were then suspended in a PBS solution with 10 wt% PVP. The measurements were carried out within 3 hours after the preparation of these RBCs. By this treatment, RBCs of 0.25 ≤ *DI* ≤ 0.6 were obtained during the measurement.

As mentioned above, PVP was mixed into each solution in order to increase the shear stress of the flow. Kolin *et al.* [17] indicated that a shear stress of 15 Pa is required to deform a normal RBC to the point that is exhibits a deformation index of *DI* = 0.5. Because this result could not be obtained in the present microchannel using only the PBS solution, PVP (MW = 3.6 × 10^5^, Nacalai Tesque; K-90) was mixed with the PBS. The viscosity of the solution as measured by a rheometer yielded a value of 0.08–0.09 Pa·s, which was approximately 100 times greater than that of the PBS solution alone.

## 4. Results and Discussion

### 4.1. Numerical Results

**Figure 4.**
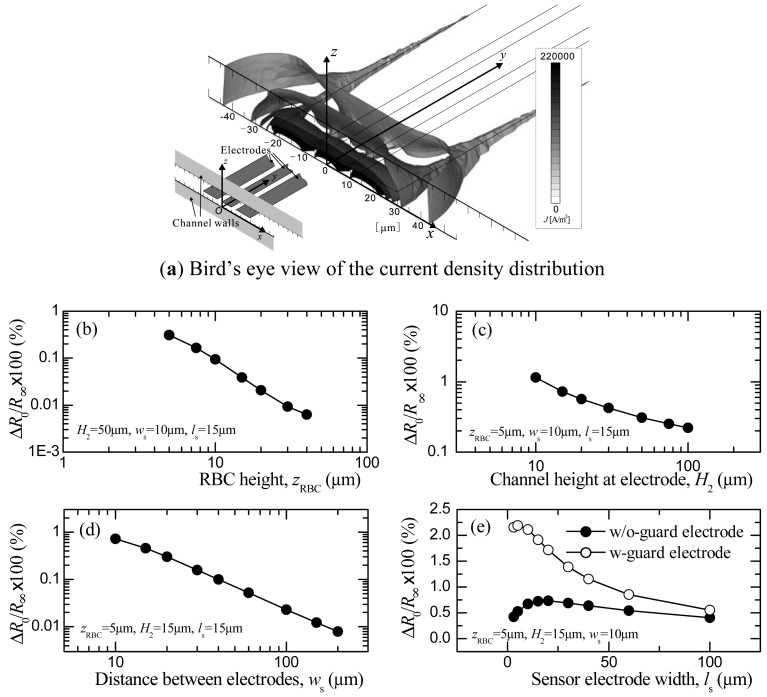
(**a**) Current density distributions around the electrodes; (**b–e**) effects of the geometric parameter of the microchannel and electrodes on the sensor sensitivity in the case of *x*_RBC_ = 0 (simulation).

The effects of the RBC height *z*_RBC_, channel height *H*_2_, distance between the sensor and ground electrodes *w*_s_, and width of the sensor electrode *l*_s_ on the electric resistance *R* will be discussed in this section. Here, we define the resistance *R*_x_ as the value obtained when the RBC is at the streamwise location *x*_RBC_. In addition, when *x*_RBC_ = 0, *R*_x_ is defined as *R*_0_, and for *x*_RBC_→∞, *R*_x_ is *R*_∞_. Naturally, *R*_0_ will be the maximum value of *R*_x_. On the other hand, *R*_∞_ will be the resistance of the solution alone and is referred to as the base resistance. The maximum value of the resistance variation, therefore, becomes Δ*R*_0_ = *R*_0_ − *R*_∞_. Since the sensitivity of the sensor depends on the degree of Δ*R*_0_/*R*_∞_, the relationships between each of the geometric parameters of the sensor and Δ*R*_0_/*R*_∞_ are shown in Figure 4(b–e).

In order to increase Δ*R*_0_/*R*_∞_, the RBC must pass through an area of relatively high current density. To be more precise, the ratio between the current density that has been interrupted by the RBC and the one penetrating the residual area should be increased in order to enlarge Δ*R/R*_∞_. To provide some insight into the features of the current density distribution as background for the following discussion, a contour map of the current density obtained under the condition of the optimized geometry is presented in Figure 4(a). As can be seen in the figure, the current flux is mainly produced by the edges of the electrodes. High current density distribution is obtained at the front edge of the sensor electrode and decreases markedly along the *z* axis.

Figure 4(b–e) shows how *z*_RBC_, *H*_2_, *w*_s_, and *l*_s_ influence the Δ*R*_0_/*R*_∞_ distribution. First, the relationship between *z*_RBC_ and Δ*R*_0_/*R*_∞_ is shown in Figure 4(b). As *z*_RBC_ decreases, Δ*R*_0_/*R*_∞_ increases. When considering a current density distribution between two-point electrical charges as an extreme condition, the current density will attenuate by a square function in relation to the distance from the two-point charges. We expected to observe a similar distribution in the electrodes used in this study, as shown in Figure 4(a). That is, a high current density would be produced between the spanwise edges of the sensor and the ground electrodes, and the current density would largely decrease with distance from the electrodes in the streamwise direction and, in particular, in the height direction. A smaller *z*_RBC_ will increase Δ*R*_0_/*R*_∞_ and is preferable, considering the sensitivity of the sensor. However, since a RBC has a certain diameter (for example, the diameter of a RBC in the simulation is a maximum of *d*_RBC_ = 6.7 μm) there is a limitation in positioning a RBC close to the channel bottom wall. For this reason, *z*_RBC_ is set at *z*_RBC_ = 5 μm for our discussion of the remaining variables.

As shown in Figure 4(c), Δ*R*_0_/*R*_∞_ increases as *H*_2_ decreases, a result we believe can be explained to the following. The current density in the area that is interrupted by the RBC will not be affected much by *H*_2_. On the other hand, the residual area decreases as *H*_2_ decreases. The ratio between these two areas increases when *H*_2_ is small, which leads to an increase in Δ*R/R*_∞_. A smaller *H*_2_ is therefore preferable from the point of view of increasing the sensor sensitivity. However, when *H*_2_ is equivalent to the RBC diameter, the RBC then assumes a parachute shape due to the Poiseuille flow velocity distribution generated in the channel. Since deformability measurement cannot be made in this case, *H*_2_ = 15 μm is chosen in the computation.

Δ*R*_0_/*R*_∞_ increases as *w*_s_ decreases, at least in the range of 10 ≤ *w*_s_ ≤ 150 μm, as shown in Figure 4(d). When the distance between the sensor and ground electrodes deceases, the current density in the area where the RBC passes increases, leading to an increase in Δ*R*_0_/*R*_∞_. The *w*_s_ that shows the largest value *w*_s_ = 10 μm is chosen in this study.

In Figure 4(e), the relationship between *l*_s_ and Δ*R*_0_/*R*_∞_, and the results for the sensors with and without guard electrodes are both depicted. In the case without the guard electrode, Δ*R/R*_∞_ assumes a maximum value near *l*_s_ = 20 μm. When *l*_s_ is larger than the diameter of the RBC, the current density distribution occupies a wide area that makes the influence of the RBC small. On the other hand, when *l*_s_ is very small, the current density is concentrated in the area close to the channel bottom wall while that along the fringe still exists. Therefore, the area interrupted by the RBC decreases, and Δ*R/R*_∞_ decreases.

In the case of guard electrodes, the current fluxes along the fringe are suppressed because of the equivalent potential field generated on both sides of the sensor electrodes. Since the fringe can be considered as an additional area that has current fluxes, the total area of the current flux will decrease. This leads to an increase ino Δ*R/R*_∞_, which can be seen in the figure where Δ*R/R*_∞_ is three times larger than that obtained without guard electrodes.

Based on these results, the following set of values is considered to represent optimized dimensions for measuring the resistance of the RBC: *H*_2_ = 15 μm, *w*_s_ = 10 μm, *l*_s_ = 10 μm, *z*_RBC_ = 5 μm.

Next, to evaluate the relationship between the deformation index *DI* and the time-series distribution of the resistance, the electric field was calculated by varying the streamwise position of the RBC, *x*_RBC_. The optimized parameters obtained in the previous discussion are used in this case for the channel and sensor geometry. Since the resistance distribution will be symmetric to the *x*_RBC_ = 0, calculation was carried out for the conditions of *x*_RBC_ ≥ 0. RBCs with different shapes were calculated by varying *DI* in the range of 0 < *DI* < 0.5.

**Figure 5.**
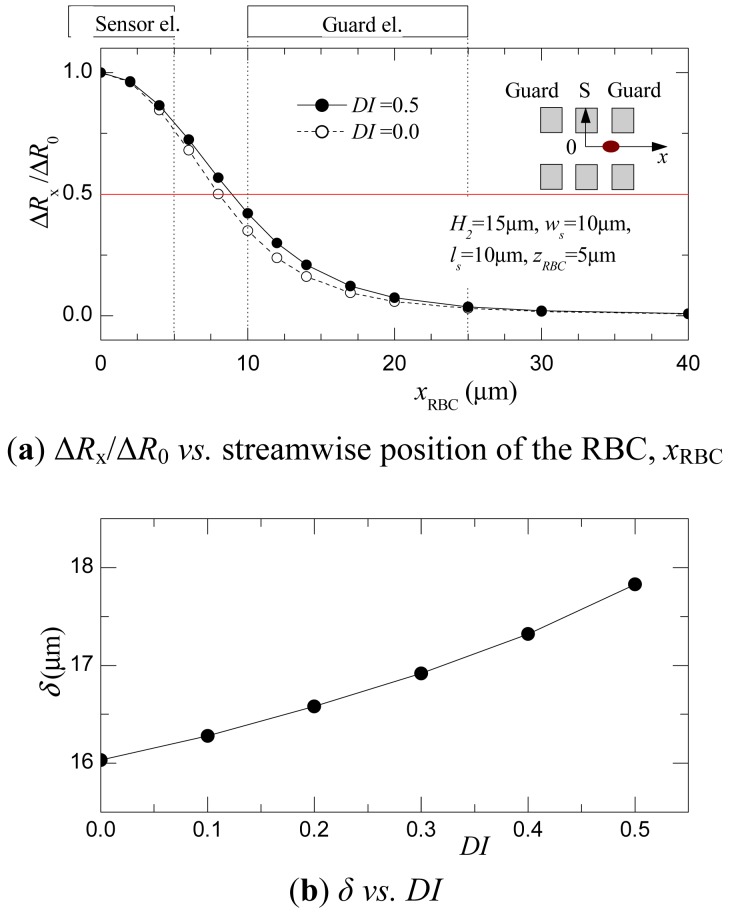
(**a**) Resistance distribution Δ*R*_x_/Δ*R*_0_ as the RBC passes between the electrodes; (**b**) the relationship between the half-bandwidth of Δ*R*_x_/Δ*R*_0_, *δ*, and the deformation index *DI* (simulation).

Figure 5(a) shows the relationship between the *x*_RBC_ and Δ*R*_x_/Δ*R*_0_ that was observed in the cases of *DI* = 0 and 0.5. Here, Δ*R*_x_, which is defined as Δ*R*_x_ = *R*_x_ − *R*_∞_, is normalized with Δ*R*_0_ (= *R*_0_ − *R*_∞_). In both *DI* cases, Δ*R*_x_/Δ*R*_0_ becomes a maximum at *x*_RBC_ = 0. Δ*R*_x_/Δ*R*_0_, then, will decrease as |*x*_RBC_| increases. Furthermore, comparing the cases of *DI* = 0 and 0.5, a broader distribution is observed in the case of *DI* = 0.5. This is attributed to fact that an ellipsoidal RBC possesses a larger streamwise length, *a*.

As an index to evaluate the deformability of a RBC from the resistance distribution, the half bandwidth of the Δ*R*_x_/Δ*R*_0_ distribution, *δ*, is calculated in each case of *DI*. The relationship of *δ* to *DI* is shown in Figure 5(b). *δ* increases monotonically with *DI* in the range of 0 ≤ *DI* ≤ 0.5. This indicates that *δ* is unique to *DI*. Therefore, deformability can be evaluated by measuring the Δ*R*_x_/Δ*R*_0_ distribution as the RBC passes between the electrodes, then analyzing it using the half bandwidth *δ*. The feasibility and validity of this method will be demonstrated experimentally in the following section. It should be noted that the difference of the half bandwidth *δ* of the ellipsoidal and spherical RBCs is smaller than the difference of their streamwise length *a*. The electric resistance is influenced by the cross-sectional of the RBC in the spanwise direction which is interrupting the electric current field, and the original current distributions the electrode is providing. In this case, the distribution shown in Figure 5 cannot be characterized by the streamwise length only, but also by the cross-sectional shape and the electrode width *l*_s_. The difference of the *δ* will increase for smaller *l*_s_ and becomes closer to the difference of the streamwise length of the RBCs. However, as *l*_s_ become smaller than a certain value the resistance decreases as shown in Figure 4(e), and the sensor signal sensitivity will deteriorate.

### 4.2. Experimental Results

Based on results of the numerical simulation, the size of the electrodes and the channel height are then defined, as shown in Table 2. There is an observable degree of difference between the values of the actual sensor and the values shown in Table 1. This is attributed to errors in the lithography process, etching process, and platinum plating, whose thickness was difficult to precisely control. For example, *w*_s_ in the numerical simulation was *w*_s_ = 10 μm, while the value shown in Table 2 is *w*_s_ = 5.2 μm. The design of the electrode width was 10 μm when spattered on the substrate. In order to plate the nano platinum particles on the electrode surface a certain time was required which particularly increased the thickness of the edges and decreased *w*_s_.

**Figure 6.**
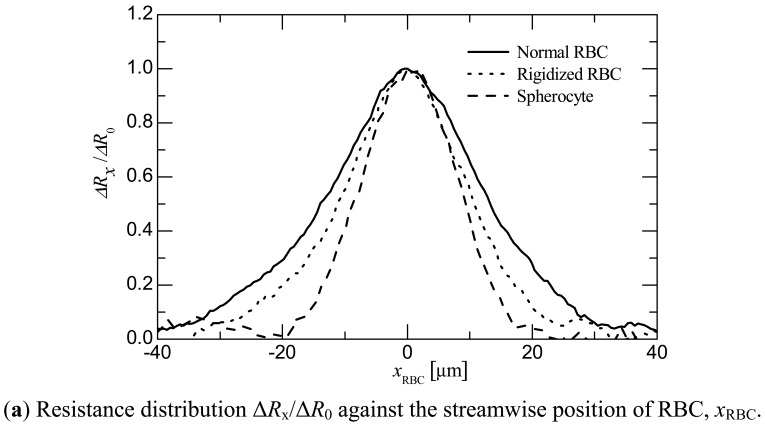

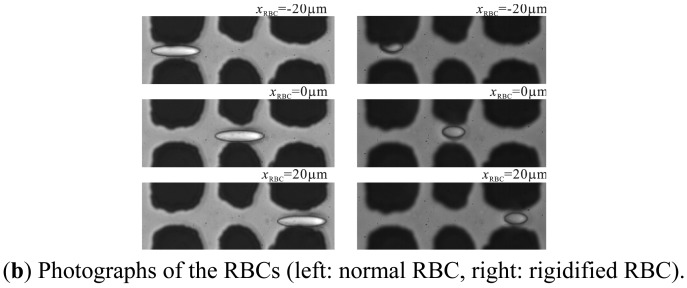
(**a**) Relationship between the normalized resistance distribution Δ*R*_x_/Δ*R*_0_ against the streamwise position of RBC, *x*_RBC_, for normal RBC, rigidified RBC and spherocyte; (**b**) photographs of normal and rigidified RBCs passing between the electrodes.

Figure 6(a) shows the distribution of the resistance ratio Δ*R_x_*/Δ*R*_0_ at the moment when the RBC passed between the electrodes. The abscissa *x*_RBC_ is the streamwise position of the RBC. *x*_RBC_ was obtained by multiplying the time recorded by the electrical measurement by the streamwise velocity of the RBC as measured by the high-speed camera. Δ*R_x_*, Δ*R*_0_, and Δ*R*_∞_ are defined in the same way as in Section 4.1. The results that represent the distributions of the normal RBC, rigidified RBC, and spherocytes are shown in the figure. In addition to this, photographs of a normal RBC and rigidified one passing between the electrodes are shown in Figure 6(b). This result corresponds exactly with that of a RBC shown in the Δ*R_x_*/Δ*R*_0_ graph. One can see in the photographs that the RBC is stretched in a streamwise direction by the shear flow and maintains its ellipsoidal shape while passing between the electrodes.

The sample numbers of the normal, rigidified, and spherical (possibly spherocytes) RBCs were 57, 30, and 8, respectively. The average *DI* and its standard deviation from that of a normal RBC were 0.56 and 0.060, respectively. On the other hand, the average and standard deviation of *DI* in the case of the spherocytes were 0.020 and 0.061, respectively. It should be noted that a variation in *DI* was observed in the case of the normal RBC, which will be addressed in the following discussion. This can be accounted for by the difference in deformability among individual RBCs, which may be associated with the effects of aging [22]. It is therefore more practical to consider the samples of normal and rigidified RBCs as one group in which there can differences in deformability.

**Figure 7.**
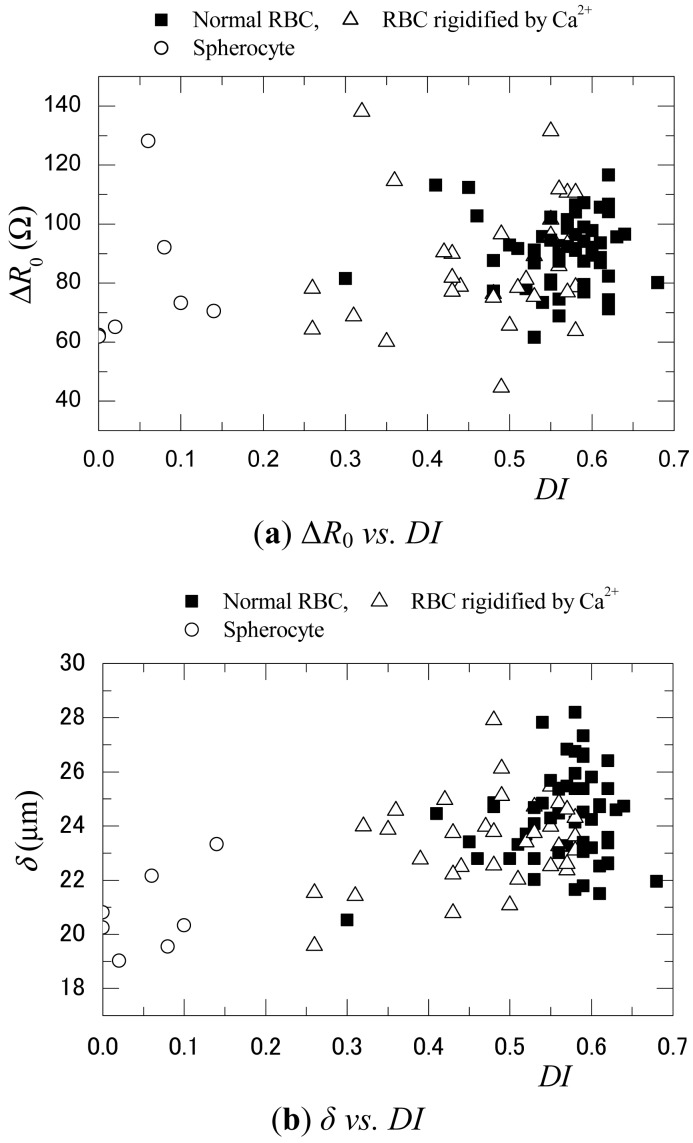
Relationship of Δ*R*_0_ and *δ* with the deformation index *DI* (experiment).

Figure 7 shows the relationship of the maximum resistance difference Δ*R*_0_ and the half bandwidth of the Δ*R*_x_/Δ*R*_0_ distribution, *δ, versus DI*. These values were obtained from the electrical and visualization measurements. It should be noted that a linear correction was made for the Δ*R*_0_ and *δ* in consideration of the spanwise position of the RBC when it passed between the electrodes. That is, as was also observed in the photographs shown in Figure 6, the spanwise position of the RBC varied in the range of −2.5 ≤ *y*_RBC_ ≤ 2.5. Although the electrodes and the RBC had a symmetric shape, Δ*R*_0_ and *δ* increased and decreased slightly depending on the spanwise position of the RBC. It is believed that the electrodes were not absolutely symmetric due to their platinum black plating, so that an asymmetric electric field was generated between them. To account for this effect, the linear components in each distribution were subtracted from each value. The results shown in Figure 7 reflect these adjustments that were made.

In Figure 7(a), it is difficult to find a correlation between Δ*R*_0_ and *DI*. Furthermore, the variation of Δ*R*_0_ is large relative to the average value. This is believed to be related to the influence of the variations in the RBC height location *z*_RBC_. As shown in Figure 4(b), the resistance shows a high sensitivity to *z*_RBC_. For example, Δ*R*_0_/*R*_∞_ varies by more than 30% when *z*_RBC_ changes from 5 μm to 10 μm. As shown in Table 1, *H*_2_, which is the height of the inlet channel for the RBC, is *H*_2_ = 10 μm, a value that is larger than the diameter of the RBC. Therefore, although the backward-facing step and the forward-facing step in the microchannel serve to make *z*_RBC_ constant, there remains some degree of variation and uncertainty in the *z*_RBC_ (≅2 μm which is calculated on the basis of the focusing depth). This error could be comparable with the sensitivity of the sensor to the differences in the RBC shape.

On the other hand, a correlation between *δ* and *DI* can be observed in Figure 7(b). Focusing on the normal and rigidified RBCs, one can see that *δ* decreases as *DI* decreases. This corresponds to the numerical results shown in Figure 5(b). Furthermore, *δ* shows a smaller value for the groups of spherocytes that possess *DI* of 0. *δ* is an index that represents the shape of the Δ*R*_x_ distribution. Therefore, compared to the case of Δ*R*_0_, *δ* depends more on the pattern of the current density distribution in the streamwise direction than on the distribution in the height direction. If the *z*_RBC_ does not fluctuate in relation to the streamwise position of the RBC, *δ* can be expected to be less affected by the current density distribution in the height direction and the *z*_RBC_. These results indicate that *δ* is an index that is more suitable for identifying RBC deformation than Δ*R*_0_.

In both figures, the scattering patterns of Δ*R*_0_ and *δ* show some errors. This can be attributed to variations in *z*_RBC_, the spanwise position of the RBC, and the size of the RBC. In particular, the spherocytes observed during measurement had a smaller volume and surface area, which would obviously influence the Δ*R*_x_ distribution in addition to the *DI* effect. To address these problems, modifications of the sensor, such as changing the shape and position of the electrodes, must be considered as future work. In any case, it can be concluded that microchannels and microelectric sensors can be used to measure the deformability of RBCs and *δ* can be used as an index to evaluate the deformation rate of the RBCs.

## 5. Conclusions

A microsensor consisting of a microchannel and micro-membrane-type electrodes was proposed, and used for measuring the deformability of a single RBC subjected to high shear flow when passing between the electrodes by analyzing the resistance distribution. The fundamental characteristics and optimal design of this apparatus were assessed by performing a numerical simulation. The performance of the sensor was then experimentally evaluated by comparing the simulation results with the results of visualization measurement carried out simultaneously with the resistance measurement. The main results were as follows:

1. A three-dimensional numerical simulation of the electric field in the region between the electrodes and in the channel wall was performed using an equivalent circuit model for the RBC cytomembrane and cytoplasm. The influences of the RBC height position, channel height, distance between the electrodes, and sensor width on the electric resistance measured using the electrodes were evaluated. Some insights into the optimal design of the sensor were obtained from these results.
2. The resistance distribution as the RBC passed between the electrodes was calculated by changing the streamwise position of the RBC. The effects of the RBC shape on the distribution pattern were investigated by changing the deformation index *DI* of the RBC. The half bandwidth of the resistance distribution, *δ*, increased monotonically with *DI*. This result shows that the deformability of RBCs can be measured by using *δ* as an evaluation index.
3. The microsensor was designed and fabricated on the basis of the numerical results. Measurements were carried out using samples of normal RBCs, Ca^2+^–A23187 rigidified RBCs, and spherocytes. The images of the RBCs were recorded using a high-speed camera. The correlation between the maximum peak of the electric resistance and *DI* was poor. On the other hand, the half bandwidth of the time-series distribution of the resistance, *δ*, showed a reasonable correlation with *DI*. In other words, *δ* increased as *DI* increased. These results corresponded to the numerical results. Thus, the feasibility of using the present sensor to measure the deformability of RBCs on the basis of microchannel shear flow was proved.

## Figures and Tables

**Table 1.** Dimensions of the sensor (μm) in the numerical simulation.

***L*_CD_**	***W*_CD_**	***H*_PDMS_**	***H*_Glass_**	***W***	***l*_sg_**	***l*_g_**
800	700	100	100	1,000	5	15

**Table 2.** Dimensions of the sensor (μm) used in the experiment.

***W***	***H*_1_**	***H*_2_**	***w*_s_**	***l*_s_**	***l*_sg_**	***l*_g_**
1,000	52	10	5.2	14.8	2.3	14.6
